# CFTR and ClC-3 Transport Fluoride Differently and Cause Dental Fluorosis in Different Ways

**DOI:** 10.3390/biom16070982

**Published:** 2026-07-03

**Authors:** Yanli Zhang, Songya Mao, Xuan Wen, Zhenxia Liu, Ying Hao, Xiaohong Duan

**Affiliations:** State Key Laboratory of Oral & Maxillofacial Reconstruction and Regeneration, National Clinical Research Center for Oral Disease, Shaanxi Key Laboratory of Stomatology, Department of Oral Biology, Clinic of Oral Rare Diseases and Genetic Diseases, School of Stomatology, The Fourth Military Medical University, Xi’an 710032, China; yanlizhang@fmmu.edu.cn (Y.Z.); sy.mao@foxmail.com (S.M.); 15009261059@163.com (X.W.); liuzhx0716@126.com (Z.L.); haoying7ice@163.com (Y.H.)

**Keywords:** fluoride, chloride, CFTR, ClC-3, genetic, dental fluorosis

## Abstract

Dental fluorosis (DF) is a common endemic disease that damages dental enamel. Traditionally, DF has been attributed to environmental fluoride overload. Accumulating evidence has demonstrated that genetic factors also modulate individual susceptibility. No dedicated fluoride ion channels have been identified in mammalian cells; fluoride uptake is believed to occur mainly through passive diffusion of HF and nonspecific anion pathways, including chloride channels. Different types of chloride channels are expressed in dental tissues, such as CFTR and voltage-gated chloride channels (ClCs), but it remains unknown whether these channels transport fluoride and whether their variants influence DF risk. This study combined human population-based investigations, mouse and zebrafish models, and in vitro experiments to confirm the significant genetic association of *CFTR* and *CLCN3* variants with DF. A total of 889 DF cases and 834 matched controls were recruited from the same fluoride-contaminated region. Tag SNP screening of *CFTR* and eight ClC chloride channel genes (*CLCNs*) revealed that rs213950 in *CFTR* and three SNPs in *CLCN3* were significantly associated with DF. CFTR and ClC-3 showed different fluoride tolerances. rs213950 in *CFTR* affected the efficiency of fluoride ion transport in Xenopus oocytes. ClC-3 enabled yeast cells to resist fluoride toxicity, whereas *clcn3* deficiency disrupted tooth and craniofacial development in zebrafish. Fluoride exposure altered nucleoprotein binding to the rs10520161 region and changed the mRNA levels of various ClC-3 transcripts. These transcripts displayed different subcellular locations and fluoride conductances and acted synergistically to confer fluoride resistance. Together, these findings raise the possibility that variants in *CFTR* and *CLCN3* may act synergistically to influence DF susceptibility. This potential interplay highlights DF as a complex trait involving dysregulated fluoride handling and underscores the multifactorial, gene-directed regulation of fluoride transport.

## 1. Introduction

As an abundant trace element, fluoride replaces the hydroxyl group in hydroxyapatite within calcified tissues and is widely used as an additive in oral hygiene products. However, fluoride has also been shown to act as a double-edged sword; excessive fluoride intake can lead to dental fluorosis (DF), skeletal fluorosis, or other adverse biological effects. Despite extensive research on the toxic effects of fluoride overdose in human and animal tissues, the specific molecular mechanisms or genes that confer resistance to fluoride toxicity in humans and other mammals remain largely unknown [1,2].

Millimolar concentrations of fluoride ions inhibit bacterial growth. Numerous bacterial and archaeal species encode fluoride-sensing RNAs that regulate the expression of proteins mitigating the deleterious effects of this anion [3]. One of the representatives is the crcB and the riboswitch-controlled ClC antiporters, which exhibit high anion selectivity for F^−^ over Cl^−^ [4,5]. The fluoride resistance of eukaryotic homologs of crcB (FEX) is further demonstrated in the filamentous fungus Neurospora crassa, the budding yeast *S. cerevisiae*, and the pathogen Candida albicans [6,7]. Whether eukaryotic voltage-gated chloride channels (ClCs) also have fluoride resistance remains unknown. These ClC channels are abundant in dental and skeletal tissues [8,9,10], yet their potential role in mediating fluoride resistance in ameloblast cells has not been determined. Meanwhile, mutations in the cystic fibrosis transmembrane conductance regulator (CFTR) gene cause cystic fibrosis (CF). Both CF and DF teeth lead to incomplete removal of enamel matrix proteins in mature enamel. Previously, we found that fluoride competed with chloride through CFTR in ameloblasts, but the CFTR siRNA treatment only blocked part of fluoride transport [11]. Thus, we asked whether the specific ClC channels and CFTR are novel susceptibility genes for DF and whether their variants contribute to the disease.

In this study, we screened for variants in *CFTR* and *CLCNs* in the Chinese population with or without DF and demonstrated that some of them act as fluoride-sensitive channels, with their variants contributing to DF susceptibility through distinct mechanisms.

## 2. Materials and Methods

### 2.1. Human Survey

#### 2.1.1. Survey Procedure

The 1723 participants were recruited from the three city/county-level areas in Shaanxi Province, China, where fluoride contamination has been monitored by the local CDC. According to local CDC surveillance data, fluoride exposure in these areas was primarily attributed to indoor combustion of fluoride-rich coal. Field measurements showed that fluoride concentrations in drinking water were below 1.0 mg/L across all study areas. All the participants in this study have lived in these areas since they were born. Individuals who had moved to the area after the age of seven or presented congenital dental anomalies were excluded. The DF phenotype was assessed based on the Dean clinical diagnostic criteria, as modified according to Chinese standards [12]; all observers were qualified and trained in the application of these criteria. For each participant, at least two observers examined the participant’s teeth and assigned the DF grade, which was subsequently reviewed by a third clinician. Ultimately, 889 DF cases and 834 matched controls were included in the following study, and their demographic information, such as age, height, weight, residence, family history and medical history, was also included. Cases and controls were frequency-matched by location, ethnicity, age and gender (Supplementary Materials Table S1). The privacy rights of participants have been observed and their written informed consents were obtained before the studies. All the work was carried out in accordance with the World Medical Association Declaration of Helsinki and the procedures have been approved by the IRB of the School of Stomatology, the Fourth Military Medical University (2009K17-06; 81070819), approval dates: 2 March 2009; 2 March 2009.

#### 2.1.2. Sample Collections and Gene Analysis

The genomic DNA was extracted from the participants’ peripheral blood following standard protocols and subsequently used for haplotype-tagging SNP analysis on the microAssay platform. Haplotype tag SNPs of eight ClC channels (*CLCN1~7*, *CLCNKB*) and *CFTR* were selected using the Genome Variation Server (GVS; University of Washington), a web-based tool that was publicly available at the time the study was conducted. Tag SNPs were selected based on linkage disequilibrium information from Asian populations available in the database. Primer information is provided in Supplementary Materials Table S2. Genotyping quality control was performed using predefined criteria, including SNP and sample call rate thresholds of 95% and a minor allele frequency cutoff of 10%. The Hardy–Weinberg equilibrium (HWE) was assessed in the control group for all autosomal SNPs as a genotyping quality-control procedure. SNPs located in *CLCN4* and *CLCN5* were excluded from HWE evaluation because both genes are located on the X chromosome. Association analyses for autosomal SNPs were performed using Pearson’s χ^2^ test and logistic regression models (Supplementary Materials Table S3). Multivariable logistic regression analysis was performed to analyze the covariates such as sex and place of residence (Supplementary Materials Table S4).

The relationship between the rs213950 genotype and DF phenotype was further investigated in a four-generation DF family from a fourth, geographically distinct location. The rs213950 genotypes of all family members were confirmed by conventional PCR followed by Sanger sequencing.

### 2.2. Mouse DF Models

#### 2.2.1. Development of Animal Model

Weanling (3-week-old) C57Black/6 mice were obtained from the Animal Center of the Fourth Military Medical University (FMMU). All animals were maintained on a 12/12 h light/dark cycle with an ambient temperature of 21 °C. Mice were fed a constant nutrition lab diet. Mice were provided with drinking water containing 100 mg/L sodium fluoride (NaF; Sigma, Cat. No. 201154, St. Louis, MO, USA) for 3 months to induce DF [13,14]. All solutions were freshly prepared using deionized water. Control mice received deionized water alone and the baseline fluoride concentration in the drinking water was below 0.1 mg/L. A total of 20 mice were used in this study (*n* = 10 per group for biochemical and molecular analyses). All animal experiments were conducted in strict accordance with the ARRIVE 2.0 guidelines to ensure comprehensive reporting of study design, methods, and results. Experimental procedures involving all mice and the subsequent zebrafish studies were approved by the IACUC of the School of Stomatology, the Fourth Military Medical University (81771052 (2017-15, 2017-16), 82100958 (2021-041)), approval date(s): 1 March 2017; 4 March 2021.

#### 2.2.2. Histological and Immunohistochemical Analysis

The tooth samples were collected and fixed in 4% paraformaldehyde in a 0.01 mol/L phosphate buffer (pH 7.4). After fixation, the tissues were processed by decalcification in 10% ethylene-diamine tetraacetic acid (EDTA; pH 7.4; Sigma-Aldrich, St. Louis, MO, USA) for about 20 days at 37 °C. The specimens were dehydrated and embedded in paraffin and the sections were stained with H&E in the regular way. Regular SABC (Streptavidin–Biotin Complex) immunohistochemistry staining (Boster, Wuhan, China) was used to detect the CLC-3 distribution with rabbit anti-CLC-3 antibody (LSBio, Newark, CA, USA). Negative controls were prepared by omitting the primary antibody.

### 2.3. Zebrafish Experiment

#### 2.3.1. Zebrafish Maintenance

Husbandry, breeding and housing conditions of the embryos and adult AB zebrafish are described in detail in our previous report [15,16].

#### 2.3.2. Morpholino Injection

The antisense morpholino oligonucleotides (MOs) were purchased from Gene Tools (Eugene, OR, USA). The *clcn3* MO with the sequence (5′-CAC CCT CTG CGA TGC CTC GAA ACG A-3′), which was a translation blocking MO (tbMO) targeting -13 to -37 in exon 1. The sequence of standard control MO was 5′-CCT CTT ACC TCA GTT AC AAT TTA TA-3′. An amount of 1 nl of a 0.475 nM *clcn3* MO was injected into zebrafish embryos at the one-cell stage into the yolk. The dose used in our experiments was the lowest one leading to the supposedly specific phenotype in 75% of embryos.

#### 2.3.3. Fluoride Treatment

Embryos injected with MO and control embryos were maintained in embryo medium containing either 0, 38, or 78 ppm NaF (NaF; Sigma, No. 201154) until 5 dpf (days post-fertilization), when they were collected for morphological and transcript analyses.

#### 2.3.4. Morphological and Histological Analysis

The general phenotypes were systematically analyzed under a Leica M205 FC stereomicroscope (Leica Microsystems Nussloch GmbH, Heidelberger, Germany). For the characterization of tooth and craniofacial structures, 5 dpf zebrafish were stained with whole-mount Alcian blue and Alizarin red as previously described [15]. Determined parameters included the length of body, head, ceratohyal and palatoquadrat; the width of head; the distance between Meckel’s cartilage and ceratohyal; and the ceratohyal angle. After being stained with Alcian blue and Alizarin red, the tooth and 5th ceratobranchial arches (5th Cb) were isolated for the ultrastructure analysis using a scanning electron microscope (SEM) (HITACHI S-4800, Hitachinaka, Ibaraki, Japan) as previously reported [16].

#### 2.3.5. Transcriptome Analysis

Total RNA was extracted using Trizol reagent (Life Technologies, Carlsbad, CA, USA) from pooled of 5 dpf and performed transcriptome analysis. High-quality RNA from each sample was combined into a single large pool in order to maximize the diversity of transcriptional units. The RNA library was constructed using Illumina’s TruSeq RNA Sample Preparation Kit following the manufacturer’s protocols (Illumina Inc., San Diego, CA, USA). The amplified flowcell was used for paired-end sequencing on a HiSeq 2000 System (TruSeq SBS KIT-HS V3, Illumina). The cluster analysis of the gene expression patterns was performed using Cluster 3.0 and the JavaTreeview software v1.1.6r4.

### 2.4. Ameloblast-like Cell LS8 Analysis

#### 2.4.1. Cell Culture

LS8 cells, an SV40-immortalized mouse ameloblast-like cell line, were kindly provided by Dr. Malcolm L. Snead at USC, Los Angeles, CA, USA. Cells were cultured in Dulbecco’s modified Eagle’s medium (DMEM; Gibco, Grand Island, NY, USA) with 10% fetal bovine serum (FBS; Gibco, Grand Island, NY, USA), 100 U/mL penicillin and 100 mg/mL streptomycin at 37 °C, 5% CO_2_ in a humidified atmosphere.

#### 2.4.2. siRNA Treatment

The small interfering RNA (siRNA) duplexes that target the mouse *Clcn3* were designed and synthesized by GenePharma according to GenBank^TM^ (AF029347) (sense, 5′-CGA GAG AAG UGU AAG GAC ATT-3′ and antisense, 5′-UGU CCU UAC ACU UCU GAA CGU ACG UTT-3′). The nonsense siRNA sequence (sense, 5′-UUC UUC GAA CGU GUC ACG UTT-3′ and antisense, 5′-ACG UGA CAC GUU CGG AGA ATT-3′) was used as a control. LS8 cells were plated at 1 × 10^4^ cells/mL, cultured in DMEM in six-well plates overnight and were transfected with siRNA duplexes using Lipofecamine 2000, and the final siRNA concentration was 100 nM. After 5 h of transfection, the growth medium was refreshed with regular growth medium.

#### 2.4.3. Fluoride Treatment and RNA Analysis

Cells with or without *Clcn3* siRNA treatment were treated with 2 mM NaCl (NaCl; Sigma, No. S7653) or 2 mM NaF (NaF; Sigma, No. 201154) for 48 h [1,17] and the total RNA was isolated for transcriptome analysis. The differentially expressed genes were further analyzed using Q-PCR methods. The *Clcn3* primers have been reported in the previous study [18]. Each set of samples was analyzed in 4 wells, and the experiments were repeated 3 times.

#### 2.4.4. Analysis of Intracellular [Cl^−^] and Fluoride Ion

[Cl^−^]_i_ is detected by the mechanism of diffusion-limited collisional quenching of MQAE fluorescence. The degree of fluorescent quenching in MQAE indicates an increase in [Cl^−^]_i_. After being treated with the *Clcn3* siRNA for 48 h, cells were incubated with 0.5 mM to 5 mM fluoride (NaF; Sigma, No. 201154) for 30 min and then the cells were incubated for 1 h at 37 °C with 5 mM MQAE in a physiological salt solution (PSS) containing 134 mM NaCl (NaCl; Sigma, No. S7653), 6 mM KCl, 2.5 mM CaCl_2_, 0.5 mM MgCl_2_, 10 mM glucose, and 10 mM HEPES was adjusted to pH 7.3 with tris-(hydroxyethyl)-aminomethane (Tris). The live cells were washed with PSS five times to remove excess dye and immediately imaged using a fluorescence microscope (Leica DM6000B, Leica Microsystems, Wetzlar, Germany) under 488 nm excitation.

To detect intracellular fluoride concentration, LS8 cells were transfected with *Clcn3* siRNA for 48 h, then incubated with Chemodosimeter 1 for 60 min at 37 °C, followed by a further 30 min incubation with 5 mM NaF (NaF; Sigma, No. 201154). Fluorescence imaging of living LS8 cells was observed under a confocal fluorescence microscope (excitation light source: 543 nm; Olympus FV1000, Olympus Corporation, Tokyo, Japan).

#### 2.4.5. Statistical Analysis

Data were presented as mean ± SD or mean ± SE. Comparisons between experimental cells and control cells were performed using an unpaired Student *t* test, and the difference was considered to be statistically significant when *p* value was less than 0.05 or 0.01. Each experiment was repeated at least three times. All researchers were fully aware of group allocation throughout the whole experiment, assessment and data analysis.

### 2.5. HEK293T Cells Experiments

#### 2.5.1. Cells and Culture

Human embryonic kidney 293T (HEK293T) cells were originally obtained from a commercial source and have been long-term stored and maintained in the laboratory. HEK293T cells were cultured in DMEM medium (HyClone, Logan, UT, USA) supplemented with 10% (*v*/*v*) fetal bovine serum (Gibco, Grand Island, NY, USA) and were maintained in 5% CO_2_ at 37 °C.

#### 2.5.2. Transcriptome Analysis

HEK293T cells were incubated with 2 mM NaCl (NaCl; Sigma, No. S7653) or 2 mM NaF (NaF; Sigma, No. 201154) in DMEM medium for 48 h. The total mRNA was extracted and used for the regular transcriptome analysis.

#### 2.5.3. ClC-3 Isoform Recombinant Lentivirus Experiment

Human isoform b (ClC-3b, NM_001829.4), isoform c (ClC-3c, NM_001243374.2) and isoform X1 (ClC-3X1, XM_005262726.3) of human *CLCN3* were synthesized and subcloned into a LV-EF1a-EGFP-target gene-PGK > Puro overexpressing system, and EGFP was used as a fused label marker before the N terminus of ClC-3 isoform. The motif LLDLLDE in ClC-3b was mutated into AADAADE in ClC-3 bmut.

HEK293 cells were infected with recombinant lentivirus under a regular process. EGFP expression in the infected cells was observed under an inverted fluorescence microscope. Puromycin was applied to set up the stable expressing cell line. Then, the recombinant lentivirus-infected cells were collected for the following experiment.

#### 2.5.4. Immunofluorescence Staining and Observing

The lentivirus-infected cells were plated in culture dishes and treated accordingly. The cells were fixed with ice cold 4% paraformaldehyde for 20 min, permeabilized with 0.25% Triton X-100 for 5 min, and then blocked with 1% BSA for 30 min at room temperature [19]. Then, the cells were stained with the following antibodies overnight: rabbit anti-GM130 polyclonal antibody (1:200, Proteintech, Chicago, IL, USA), rabbit anti-ERp72 polyclonal antibody (1:200, Proteintech, Chicago, IL, USA), and rabbit anti-AIF polyclonal antibody (1:200, Proteintech, Chicago, IL, USA). Next, it was followed by goat anti-rabbit IgG (Cy3) secondary antibody (Cowbio, Beijing, China) for 60 min at RT. Images were obtained using a laser scanning confocal microscope (Olympus FV1000, Olympus Corporation, Tokyo, Japan).

#### 2.5.5. Electrophoretic Mobility Shift Assay (EMSA)

Nuclear proteins were extracted from HEK293T cells—treated or not with 2 mM NaF (NaF; Sigma, No. 201154)—using the Nuclear and Cytoplasmic Protein Extraction Kit (CWBIO) according to the manufacturer’s instructions. The oligonucleotide probes corresponding to the native sequences (rs10520161A) (GGC AGC TAA CAA ATC GAA AAA ACT GAA TCA GCT TCC CTG TG AGG AAG AAC) and the mutated one (rs10520161T) (GGC AGC TAA CAA ATC GAA AAA ACT GTA TCA GCT TCC CTG TGA GGA AGA AC) were synthesized and labeled with biotin by Sangon Biotech (Shanghai, China). Double-strand biotin-labeled or unlabeled competitive probes were produced by annealing the labeled or unlabeled sequences, respectively. DNA gel shift assays were performed using the Chemiluminescent EMSA Kit (Beyotime, Shanghai, China). DNA binding reactions containing 15 μg of nuclear proteins, 0.2 μg of biotin-labeled probes with or without 2 μg competitive probes were performed at room temperature for 30 min. The reactions were resolved on 6% polyacrylamide gels in 0.5× Tris/borate/EDTA (TBE) buffer [20]. Gels were transferred onto a nylon membrane. Subsequently, the nylon membrane was subjected to UV crosslinking. Blots were probed with horseradish peroxidase (HRP)-conjugated streptavidin. Chemiluminescence signals were detected.

#### 2.5.6. Whole-Cell Patch Analysis

HEK293 cells overexpressing different EGFP-fused isoforms were used in the study. Chloride currents were measured at room temperature (22 °C) using standard whole-cell voltage-clamp techniques performed with an HEKA EPC10 patch-clamp amplifierdriven by PatchMaster (HEKA Elektronik Dr. Schulze GmbH, Lambrecht/Pfalz, Germany). Pipette solutions contained (in mM): 120 CsCl, 4 TEA-Cl, 5 EGTA, 1.187 CaCl_2_, 2 MgCl_2_, 5 Na-ATP, 0.5 Na_2_-GTP, 10 HEPES, pH 7.2 with CsOH. Bath solution in NaCl group contained (in mM): 120 NaCl, 1 MgCl_2_•6H_2_O, 10 HEPES, and 10 D-Glucose, pH 7.4 with NaOH. Ion substitution experiments were done by replacing NaCl with equimolar NaF in the NaF group. Osmolality of all solutions was determined using a micro OSMETTE osmometer, and all extracellular solutions were titrated to 300 mOsmol/kg H_2_O. HEPES (pKa 7.5) was used as the buffer in all experiments. All chemicals were obtained from Sigma (St. Louis, MO, USA).

Pipette resistances were 3–5 M. Pipette and whole-cell capacitance and series resistance compensations were done before recording. Identification of GFP-positive cells was done immediately before cell selection using a fluorescence-equipped inverted microscope (Micro-shot MF53). Currents were elicited from a holding potential of −40 mV to test potentials from −100 mV to +100 mV in 20 mV increments, and held for 400 ms. Currents were normalized to cell membrane capacitance and expressed as current density (pA/pF). Results are expressed as means ± SE. Unpaired Student’s *t*-tests with a Bonferroni correction were used to determine statistical significance with GraphPad Prism 8 (GraphPad Software, San Diego, CA, USA) software.

### 2.6. Oocytes Experiment

#### 2.6.1. Recombinant Vectors Construction

The recombinant EGFP-CFTR expression vector was constructed with pcDNA3 (Invitrogen Corp., San Diego, CA, USA) and the CFTR cDNA fragment (NM_00492.4) by artificial DNA synthesis. Sequencing the complete CFTR coding region of this construct did not reveal any differences except for some variants enhancing the expression in the eukaryotic system (T → C at 930, A → G at 933, T → C at 936). From this construct, an M470 and ∆F508 CFTR cDNA/pcDNA3 construct was made by means of the Transformer™ Site-directed Mutagenesis Kit (Clontech, Palo Alto, CA, USA) according to protocols provided by the manufacturer. The complete CFTR coding region of the constructs was also verified by sequencing. The key variants in the constructed vectors were as follows: M470: c.1408A; V470: c.1408G; and ∆F508: c.1520~1522delCTT).

#### 2.6.2. RNA Synthesis

CFTR transcripts were in vitro-transcribed from the CFTR cDNA (M470, V470 and ΔF508)/pcDNA3 constructs using the cRNA synthesis kit (mMESSAGE mMACHINE, Ambion Inc., Austin, TX, USA) according to the manufacturer’s protocol. Defolliculated oocytes were injected with 40 nl RNA (1 μg/μL) using a Nanoject II microinjector (Drummond Scientific, Broomall, PA, USA) with three types of CFTR cRNA, respectively.

#### 2.6.3. Oocyte Injection

Oocytes obtained from adult female toads (*Xenopus laevis*) were enzymatically defolliculated and maintained at 18 °C in modified Barth’s saline.

#### 2.6.4. Two-Electrode Voltage-Clamp Assays in Oocytes

Oocytes injected 2–3 d previously were voltage-clamped with a two-electrode technique and continuously perfused with ND96 solution (96 mM NaCl, 5 mM Hepes, 2 mM KCl, 1.8 mM CaCl_2_, 1 mM MgCl_2_; pH 7.4, 100 mM niflumic acid) to block endogenous Ca^2+^-activated Cl^−^ currents. CFTR was activated by perfusion of the oocyte with cocktail buffer containing 10 μM forskolin and 1 mM IBMX for 25 min. The oocytes being clamped were firstly perfused with (1) ND96 solution without activating cocktail buffer (Blank), next (2) with ND96 and cocktail buffer (NaCl group), and then (3) with ND96-NaF (96 mM NaCl was replaced with 96 mM NaF) and cocktail buffer (NaF group). The 96 mM NaF was applied to match the routine 96 mM NaCl concentration in oocyte electrophysiology recordings. Then, (4) with ND96 and cocktail buffer (NaF/NaCl group), and, finally, (5) with ND96 and cocktail buffer plus 10 μM CFTRinh-172 (172 group). Membrane currents in uninjected, water-injected or mock-injected oocytes were unresponsive. CFTR Cl^−^ currents were measured by voltage clamping the oocytes in 20 mV steps between −160 and +40 mV adjusted for resting transmembrane potential, and analyzed using pClamp 8.1 software (Axon Instruments, Foster City, CA, USA). To reduce error due to series resistance, the voltage clamp (Axon Geneclamp 500B, Axon Instruments, Inc., Union City, CA, USA) was configured to clamp the bath potential to 0 mV. In this configuration, we independently monitored the oocyte membrane potential during our clamp protocol and routinely observed membrane potentials that were <5% depolarized from our target holding potentials. The current–voltage (I-V) relationships from −160 mV to +40 mV were recorded and the related slopes were calculated and presented as mean ± S.E. IGO, GraphPad Prims 8 (GraphPad Software, San Diego, CA, USA) and PatchMaster (HEKA Elektronik Dr. Schulze GmbH, Lambrecht/Pfalz, Germany) were used to analyze the data.

Statistical comparisons were performed using Student’s *t* test. A pairwise *t* test was used for pre/post-treatment in experiments using an individual oocyte. A two-tailed *t* test was used when comparing currents obtained from oocytes injected with different cRNAs (i.e., M470 versus V470 or ΔF508). *p* values < 0.05 were accepted to indicate statistical significance. Statistical analyses were performed using SigmaStat version 2.03 software.

## 3. Results

### 3.1. CFTR and CLCN3 Variants Increased DF Risk

**Figure 1:**
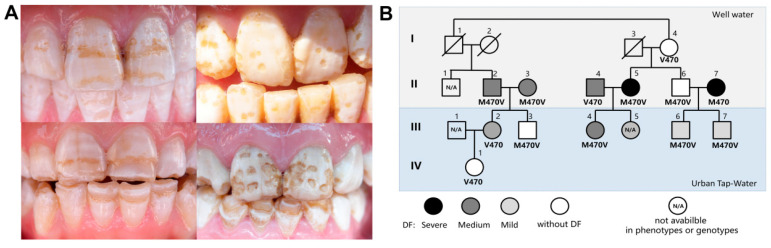
DF cases and pedigree analysis of CFTR M470V variants. (**A**) Typical tooth characteristics of DF cases in the investigation. Note enamel discoloration and local defects in the affected teeth. (**B**) DF phenotype and rs213950 genotype in a four-generation DF family. The first and second generations lived with well water and the 3rd and 4th generations lived with tap water. The five members in the 3rd generation (III_2_, III_4_, III_5_, III_6_, and III_7_) suffered from DF, although with the tap water. Numbers above the pedigree symbols indicate the individual IDs. A diagonal slash indicates a deceased individual. Black, dark gray, light gray, and white symbols represent severe, medium, mild, and unaffected dental fluorosis (DF), respectively. “N/A” indicates that the phenotype or genotype was not available.

A total of 1723 Han Chinese participants were recruited, comprising 909 males and 814 females, with a mean age of 38.0 years. Among them, 889 were diagnosed with DF, while the remaining 834 served as matched controls. All the DF cases presented with enamel discoloration and local defects to varying degrees (Figure 1A). Among the 889 DF cases, the proportions of mild, moderate, and severe DF were 47.47%, 31.05%, and 21.48%, respectively. (Supplementary Materials Table S5).

Among the 24 haplotype tag SNPs in *CFTR* and eight *CLCNs*, the three tag SNPs (rs10520161, rs17659581, and rs9996873) in the noncoding region of *CLCN3* and one SNP (rs213950) in the coding region of *CFTR* yielded evidence for association with DF (Table 1). The frequency of the c.1408G allele of the candidate susceptibility-associated variant rs213950 was markedly higher in the DF group (1.45 × 10^−42^), and its genotype was also closely correlated with DF severity (*p* = 7.97 × 10^−43^) (Supplementary Materials Table S5).

We further compared the DF phenotype and rs213950 genotype in a four-generation DF family. In this family cohort, 100% (1/1) M470, 75% (6/8) M470V and 50% (2/4) V470 presented DF. The five members in the third generation (III_2_, III_4_, III_5_, III_6_, and III_7_) suffered from DF, although living with the tap water, further suggesting that rs213950 was related to DF cases (Figure 1B).

### 3.2. V470 Variant in CFTR Is More Sensitive to Fluoride

**Figure 2:**
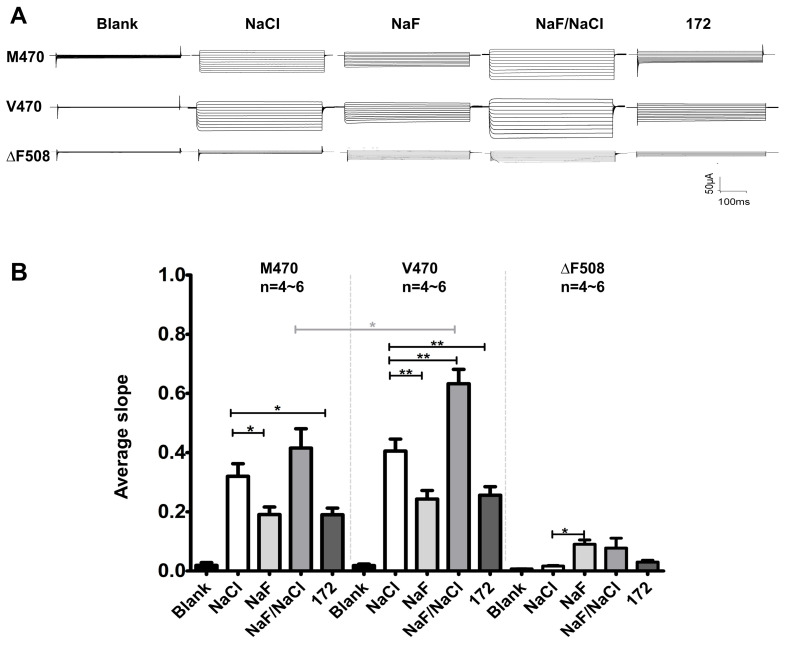
Chloride and fluoride currents of different CFTR genotypes in Xenopus oocytes. (**A**) Representative current traces of single-channel recordings of M470, V470 or ∆F508 CFTR obtained at different holding potentials in the sequence indicated. Blank: without activating cocktail containing 10 μM forskolin and 1 mM IBMX; other four groups: with activating cocktail. NaCl: ND96 solution containing 96 mM NaCl; NaF: ND96 solution containing 96 mM NaF; NaF/NaCl: transition from 96 mM NaF state to 96 mM NaCl state; 172: ND96 solution with CFTRinh172. (**B**) Comparison of currents in the above RNA-injected oocytes. The average slope was calculated to reflect the current–voltage (I-V) relationships from −160 mV to +40 mV. Both M470 and V470 showed various membrane currents under NaCl and NaF stimulating conditions. Their difference was found when 96 mM NaF was replaced by 96 mM NaCl, and the larger membrane currents were found in V470 group. CFTR (inh)-172 may inhibit the current as 96 mM NaF did. ∆F508 did not show any obvious currents in the regular ND96 current, but fluoride stimulus increased weak current. *n* = 4~6. *: *p* < 0.05; **: *p* < 0.01.

The classical two-electrode voltage-clamp recordings in Xenopus oocytes showed that both M470 and V470 groups generated large membrane currents upon 96 mM NaCl stimulation, which were completely blocked by the CFTR-specific inhibitor (CFTR (inh)-172). The ∆F508 group did not show any obvious currents in the regular ND96 current. When 96 mM NaCl in the ND96 solution was replaced with 96 mM NaF, the membrane currents in the M470 and V470 groups were markedly reduced. Upon re-application of 96 mM NaCl, the V470 group exhibited larger currents than the M470 group (Figure 2), indicating a differential fluoride tolerance between the two groups.

### 3.3. ClC-3 Is Essential for Survival from Overdose Fluoride

**Figure 3:**
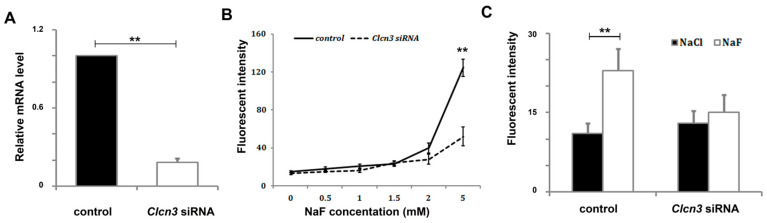
ClC-3 is necessary to prevent fluoride entrance. (**A**) Q-PCR results. Treatment with *Clcn3*-specific siRNA downregulated *Clcn3* expression by 80% in mouse ameloblast LS8 cells. (**B**) Comparison of intracellular chloride. Compared with the control, intracellular chloride levels (labeled with MQAE dye) were significantly higher in both 2 mM and 5 mM NaF-treated control groups, whereas no increase was observed in the *Clcn3* siRNA-transfected group. (**C**) Comparison of intracellular concentration of fluoride. The control group exhibited a significant increase in fluorescence after NaF treatment, whereas the *Clcn3* siRNA group showed only a weak, non-significant rise in fluoride uptake. N = 3. **: *p* < 0.01.

Mouse ameloblast LS8 cells were treated with *Clcn3*-specific siRNA, resulting in over 80% downregulation of *Clcn3* mRNA (Figure 3A). Cells from the two groups exhibited distinct intracellular chloride concentrations, as measured using MQAE dye, and the influx of chloride could be competed by 2 mM and 5 mM NaF (Figure 3B).

We then used the fluoride-sensitive dye to monitor intracellular fluoride dynamics. The control group exhibited an increased fluorescent signal after 10 min of 2 mM NaF treatment, whereas the *Clcn3* siRNA group showed no obvious fluoride uptake (Figure 3C). These results suggest that ClC-3 facilitates fluoride influx into ameloblasts.

In addition, using the yeast model, we observed that the voltage-gated chloride channel (Gef1) can help yeast against overdose fluoride (Supplementary Materials Figure S1).

### 3.4. ClC-3 Deficiency Affects Craniofacial and Tooth Development

**Figure 4:**
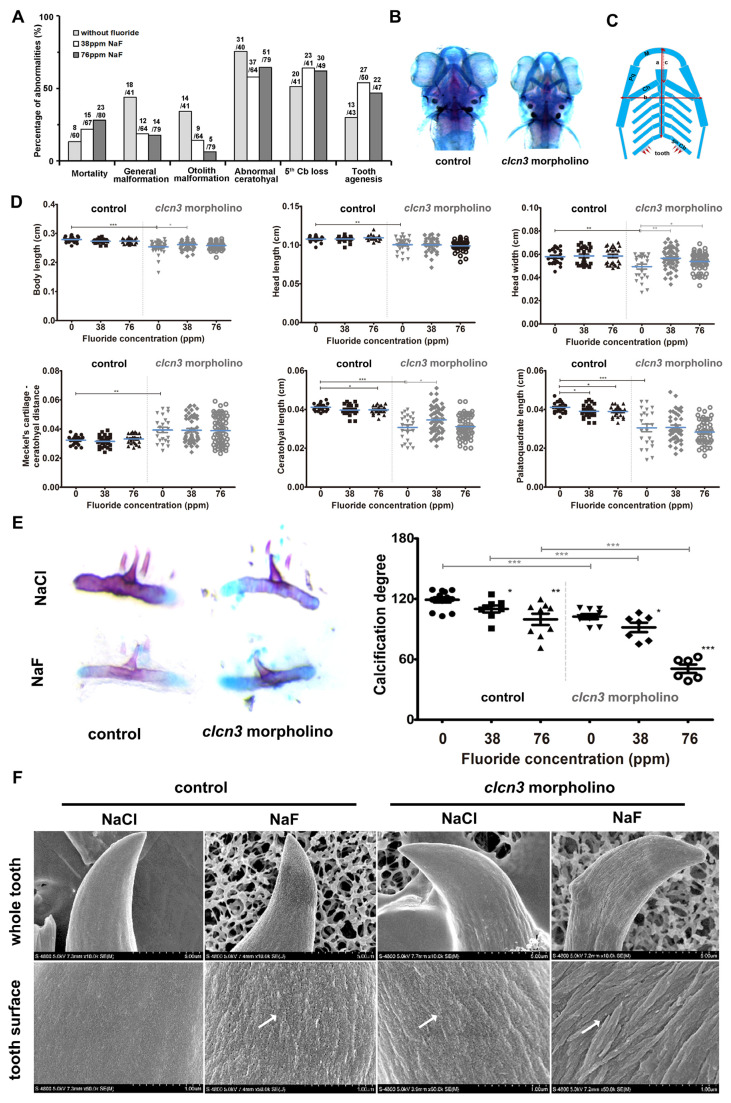
Fluoride affects the features of *clcn3* morphants. The zebrafish embryos were injected with *clcn3* morpholinos or control morpholinos and then incubated in 38 ppm or 76 ppm NaF for five days. (**A**) Ratios of abnormal phenotypes of *clcn3* morphants w/o fluoride treatment. The calculated number of zebrafish embryos was recorded in each column. (**B**) Craniofacial features of 5 dpf *clcn3* morphants using Alcian blue–Alizarin red double staining. The head skeleton of 5 dpf control zebrafish larvae presents calcified pharyngeal teeth, whereas Meckel’s cartilage and the ceratohyal remain cartilaginous. In *clcn3* morphants, the ceratohyal is malformed and the ceratohyal angle is altered. (**C**) Schematic map of zebrafish head for calculating. Ch, ceratohyal; 5th Cb, 5th ceratobranchial arches; M, Meckel’s cartilage; Pq, palatoquadrate; a, head length; b, head width; c, distance from Meckel’s cartilage to ceratohyal. (**D**) Comparison of skeleton and craniofacial parameters such as body length, head length, head width, the length of ceratohyal, Meckel’s cartilage and palatoquadrate. (**E**) Teeth characteristics of 5 dpf zebrafish larvae stained with Alcian blue–Alizarin red. The number and shape of teeth were altered in both *clcn3* morphants and fluoride-treated zebrafish embryos. Alizarin red staining intensity was markedly reduced in these groups, with the greatest decrease observed in 76 ppm NaF-treated *clcn3* morphants. (**F**) Ultrastructure characteristics of 5 dpf zebrafish teeth using SEM observation. *clcn3* morphants and fluoride-treated zebrafish embryos exhibited a rough surface on the teeth. Fluoride treatment further worsened the tooth deformities in *clcn3* morphants. White arrows point to the rough surface on enameloid surface. Scale bars represent 5 μm (upper panel) and 1 μm (lower panel), respectively. *, *p* < 0.05; **, *p* < 0.01; ***, *p* < 0.001.

The *clcn3*-specific morpholinos were designed to knock down the endogenous ClC-3 level in zebrafish. The mortality rate of *clcn3* morphants was 13.33%. Knockdown of *clcn3* resulted in body malformations (43.90%) such as curved body, cardiac abnormalities, etc. (Figure 4A). We mainly focused on the changes in skeletal systems, especially in craniofacial bones and teeth.

The typical craniofacial phenotypes of *clcn3* morphants included abnormal ceratohyal (Ch) with enlarged or inverted Ch angle (75.61%), fifth Cb absence (51.20%), the increased distance from Meckel’s cartilage (M) to ceratohyal (Ch) and shorter Ch and palatoquadrate (Pq), which may contribute to the shorter head. The *clcn3* morphants demonstrated hypocalcification of most bones and presented as weak alizarin red staining. A total of 34.10% of *clcn3* morphants lost otolith and the calcification degree of otolith was also decreased (Figure 4A–C). The 30.23% *clcn3* morphants presented tooth agenesis. Compared to the control, *clcn3* morphants presented deformed teeth with decreased calcium deposition and a rough surface (Figure 4D,E).

### 3.5. ClC-3 Impacts on the Fluoride’s Effects on Bone and Teeth

Zebrafish embryos injected with *clcn3* morpholino and their control siblings were exposed to 38 ppm or 76 ppm NaF for five days. Fluoride treatment exacerbated certain defects—such as the proportion of embryos with short, curved bodies and abnormal ceratohyal and otolith morphology—while alleviating others, including the incidence of missing fifth Cb and teeth (Figure 4A,D).

As shown in our previous work, control tooth cusps appeared red after whole-mount Alizarin red–Alcian blue staining [15], whereas exposure to 76 ppm NaF produced malformed teeth with uneven red staining. In *clcn3* morphants, 76 ppm NaF further increased the proportion of missing teeth and reduced tooth mineralization, resulting in a higher dental fluorosis index (Figure 4E,F).

### 3.6. Various ClC-3 Transcripts Respond Differently to Overdose Fluoride

**Figure 5:**
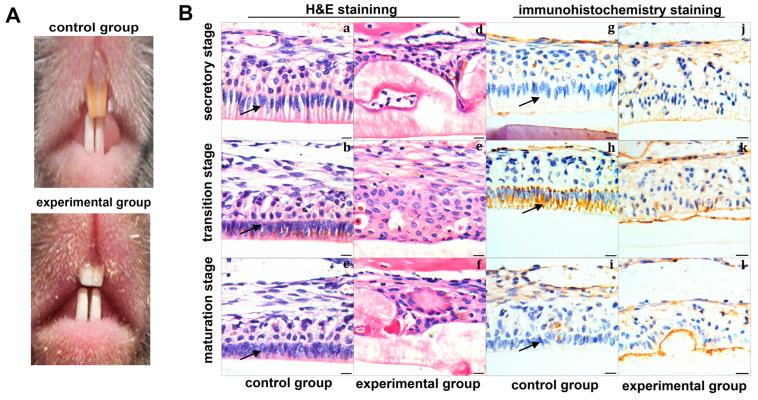
Correlation between fluoride and ClC-3 in DF mouse model. (**A**) Appearance of mouse incisors in control (upper lane) and DF mouse model (lower lane). (**B**) Histological changes in mouse incisors and the related ClC-3 changes. (a~f) H&E staining. Images a~f illustrate sequential stages of mouse mandibular incisor enamel differentiation. In the secretory stage, normal ameloblasts are tall, columnar and regularly aligned (a); they shorten during the transition (b) and maturation stages (c). In DF-treated samples, ameloblasts are irregularly arranged, cysts are present, and the enamel is malformed (d~f). (g~l): sABC immunohistochemical staining for CLC-3 in tooth. Images (h) shows strong positive staining for CLC-3 in ameloblasts at the transition stage in the control group. By contrast, positive staining for CLC-3 is observed in DF-treated ameloblasts at all three stages (j~l). Black arrows point to the ameloblasts. Scale bars = 10 μm.

Mouse DF incisors exhibited obvious fluorotic changes, including tooth discoloration, irregularly arranged ameloblasts, cysts, and malformed enamel (Figure 5A,B). Immunohistochemistry revealed fluoride-induced alterations in the abundance and distribution of CLC-3 in the tooth (Figure 5B), suggesting that ClC-3 may be associated with fluorosis-related enamel pathogenesis.

**Figure 6:**
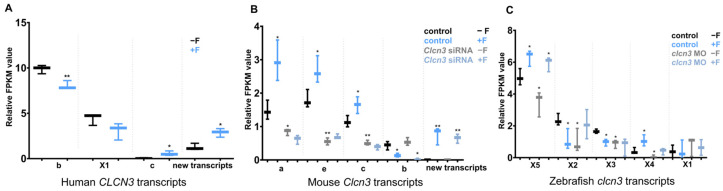
Comparison of fluoride-induced response among ClC-3 transcripts in human HEK 293 cells, mouse ameloblast LS8 cells and zebrafish embryos. (**A**) Various human *CLCN3* transcripts respond differently to fluoride. In untreated HEK 293 cells, transcripts *CLCN3b* and *CLCN3X1* were predominant, whereas *CLCN3*c and several unreported transcripts were barely detectable. After exposure to 2 mM NaF for 24 h, the levels of *CLCN3b* and *CLCN3X1* were reduced, with the decrease in *CLCN3b* being statistically significant (*p* < 0.01). In contrast, the trace amounts of *CLCN3*c and the previously unreported transcripts were markedly increased. (**B**) Comparison of different mouse *Clcn3* transcripts. Various transcripts exhibited distinct responses to excessive fluoride. The fluoride-induced changes in transcripts a, c, and e were suppressed by *Clcn3* siRNA, whereas those in transcript b were not. Excess fluoride also induced the expression of several previously unreported transcripts in both human and murine cell lines. (**C**) Comparison of different zebrafish *clcn3* transcripts. Control and *clcn3* morpholino zebrafish larvae were cultured for 48 h in 76 ppm NaCl or 76 ppm NaF. Transcriptome analysis revealed that *clcn3* X2–X5 transcript levels were reduced by the *clcn3* morpholino. These transcripts also responded differently to fluoride overdose, and the morpholino attenuated several of the fluoride-induced changes (X5, X2, X4). *: *p* < 0.05; **: *p* < 0.01.

We then employed the in vitro or in vivo models to elucidate the detailed mechanism underlying fluoride-mediated regulation of ClC-3 transcription. ClC-3 transcription is complex: human *CLCN3* is predicted to yield at least six isoforms with distinct N- and C-terminal sequences (Supplementary Materials Table S6). Transcriptome profiling indicates that human HEK 293 cells predominantly express isoforms b and X1 (Figure 6A), whereas the mouse LS8 cell line chiefly expresses three other isoforms (a, e, c). After 48 h of exposure to 2 mM NaF, mRNA levels of isoform b declined and those of isoform c rose in both cell lines (Figure 6B). Zebrafish express at least five *clcn3* transcripts (X1–X5) (Supplementary Materials Table S6). Exposure to 76 ppm NaF decreased X2 and X3 levels while increasing X4 and X5 (Figure 6C). Knock-down of X2–X5 with *clcn3* morpholino abolished the fluoride-induced changes in X2–X4 but left X5 unaffected (Figure 6C). Thus, across species, certain ClC-3 transcripts show similar fluoride-responsive up- or down-regulation.

### 3.7. ClC-3 Transcripts Selectively Transport Fluoride

**Figure 7:**
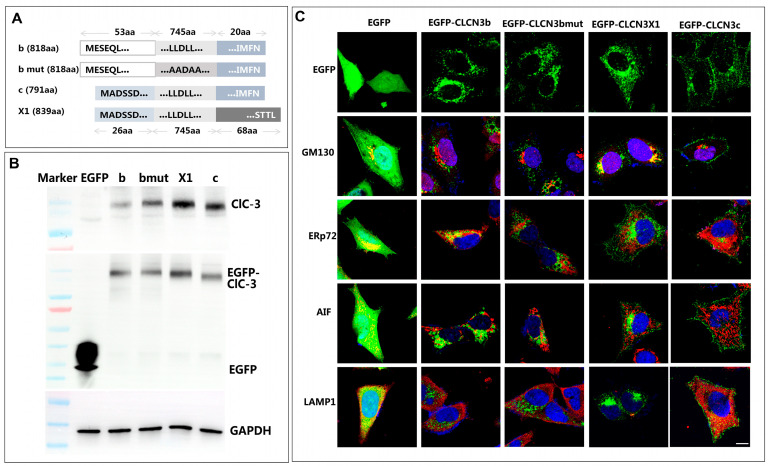
Expression of various ClC-3 isoforms in HEK293 cells. (**A**) Schematic map of various human ClC-3 transcripts and their structures. Only representative amino acid sequences are shown. Ellipses (“…”) indicate omitted amino acid residues not shown in the schematic sequence. (**B**) Western blot analysis results. The upper, middle and lower lanes were the results with antibodies against ClC-3, EGFP and GAPDH, respectively. The endogenous ClC-3 protein level was not detected in EGFP group, but the other four overexpressing groups (ClC-3b, ClC-3 bmut, ClC-3c, ClC-3X1) showed the EGFP-ClC-3 protein expression, which had minor difference in molecular size. (**C**) Confocal images. Green represented the location of overexpressed EGFP or EGFP fusion proteins. Red signals represented the intracellular staining of GM130, ERp72, AIF and LAMP1, respectively. Blue represents DAPI staining of cell nuclei. Colocalization was shown in yellow. Scale bars = 10 μm.

Because the various ClC-3 transcripts respond differently to fluoride, we sought to determine whether they also differ in expression level and subcellular localization. We used EGFP-tagged lentiviruses to individually overexpress the following human ClC-3 transcripts in HEK 293 cells: ClC-3b (NM_001829.4), ClC-3c (NM_001243374.2), ClC-3X1 (XM_005262726.3) and ClC-3b mut (NP_001820.2 p.LLDLL 71~75 AADAA) (Figure 7A). Western blot analysis revealed distinct protein levels of EGFP-fused isoforms a, b, and X1 (Figure 7B).

ClC-3b was mainly localized to intracellular vesicles and colocalized with ERp72 (an endoplasmic reticulum (ER)-lumen marker), AIF (a ubiquitous mitochondrial oxidoreductase), and partially with GM130 (a Golgi-associated peripheral membrane protein) and LAMP1 (a lysosomal marker). The LLDLL→AADAA mutation decreased the expression in ER localization, consistent with previous findings [21]. Isoforms c and X1 were predominantly found on the plasma membrane; X1 exhibited greater colocalization with GM130, ERp72 and AIF than isoform c (Figure 7C).

**Figure 8:**
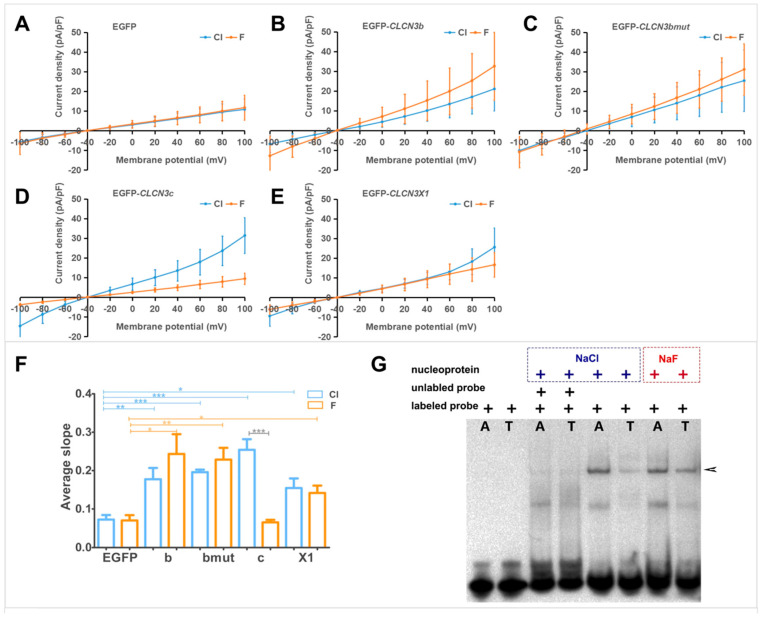
Function and regulation of different ClC-3 isoforms. (**A**–**F**) Chloride and fluoride transport properties of different ClC-3 isoforms. Whole-cell recordings were used to detect the chloride (blue curve) and fluoride (orange curve) currents in the HEK293 cells expressing ClC-3b, bmut, c, and X1. Four groups (ClC-3b, bmut, c, and X1) yielded ClC-3-specific chloride currents distinguishable from EGFP control cells and the ClC-3c group showed the highest amplitude. Fluoride current was recorded only in ClC-3b, bmut, and X1. ClC-3c group could not conduct any fluoride current (*p* < 0.001). Image (**F**) is the comparison of the average slope. *n* = 4~6. *: *p* < 0.05; **: *p* < 0.01; ***: *p* < 0.001. (**G**) EMSA results showing different binding effects of the rs10520161 A-allele or T-allele. Double-stranded DNA probes corresponding to the *CLCN3* rs10520161 A-allele and T-allele were labeled with digoxigenin and incubated with nuclear extracts from HEK-293 cells. Both probes formed shifted complexes of similar mobility. Competition with excess unlabeled allele-specific probes abolished the shifts, confirming specific binding. Band intensities revealed that the A-allele probe bound more strongly than the T-allele probe in control extracts. In contrast, after exposure to excess fluoride, the T-allele probe generated a more intense shifted band than the A-allele probe, indicating that fluoride reversed the relative binding affinity. The arrow indicates the nucleoprotein–probe complex.

Whole-cell recordings of HEK293 cells expressing human ClC-3b, ClC-3c, or ClC-3 X1 revealed isoform-specific currents that were clearly distinguishable from those in EGFP-expressing controls. All four groups (b, bmut, c, and X1) mediated chloride transport, with group c showing the highest slope. Meanwhile, three groups (b, bmut, and X1) exhibited appreciable fluoride transport, whereas group c showed none. Notably, both the b and bmut isoforms exhibited a higher slope for fluoride transport than for chloride transport. At the mid-slope point of the transport curve, ClC-3 X1 transported chloride and fluoride to the same extent, showing no significant difference between the two anions (Figure 8A–F).

### 3.8. Fluoride Affects Nucleoprotein Binding

GeneHancer (GH) Regulatory Elements annotation indicates that rs10520161 lies upstream of the canonical transcript but still within the gene body because of alternatively transcribed isoforms and is predicted to affect embryonic craniofacial development based on the craniofacial atlas. To test whether fluoride modulates ClC-3 transcription via rs10520161, we performed EMSA using nuclear extracts from HEK293 cells and biotin-labeled double-stranded probes containing either the rs10520161 A-allele or T-allele. As shown in Figure 8G, nuclear extracts from HEK293 cells retarded the migration of both A- and T-allele probes, with the T-allele showing weaker binding. Competition with excess unlabeled A- or T-allele probe abolished the shift, confirming specificity. After 2 mM NaF treatment, nuclear extracts displayed markedly enhanced binding to the T-allele probe, indicating that the T-allele is more sensitive to fluoride (Figure 8G). Of note, this is only preliminary allele-specific binding evidence, and the exact binding proteins remain unidentified.

## 4. Discussion

DF is a complex endemic disease influenced by both environmental and genetic factors [22,23]. Polymorphisms in more than 12 genes have been analyzed in DF patients and controls, comprising risk genes (*COL1A2*, *ESR2*, *DLX1*, *DLX2*, *AMBN*, *TUFT1*, *TFIP11*, *miRNA17*, and *SOD2*) and protective genes (*ESR1*, *MMP20*, and *ENAM*) [2]. Various DF characteristics were also observed in mice with different genetic backgrounds [15,24]. In this study, we found that *CFTR* and *CLCN3* are novel susceptibility genes for DF and function as protective factors in distinct ways.

One of the interesting findings was that the CFTR coding variant rs213950 (M470V) was identified as a candidate susceptibility-associated variant for DF. M470 CFTR proteins matured more slowly and had a different chloride channel activity compared with V470 CFTR proteins [25]. Here, we found that CFTR mediates fluoride transport, and the V470 mutation alters chloride conductance upon fluoride exposure. This suggests that fluoride may modulate cellular activity either through direct fluoride-mediated effects or via chloride channel-dependent mechanisms, such as impaired endocytosis and protein degradation that we previously demonstrated [11].

ClC-3 appears to be under complex regulatory control by its variants and fluoride. ClC channels exhibit low anion selectivity, transporting Cl^−^, Br^−^, I^−^, NO^−^ and SCN^−^, yet evidence for fluoride transport by these channels remains scarce. CLC-ec1 from bacteria has been reported to be highly selective for F^−^ over Cl^−^ [26,27], whereas Gef1 is a representative ClC channel in yeast [28,29]. We show here that Gef1 helps yeast resist fluoride toxicity, and overexpression of human ClC-3 restores yeast survival under excess fluoride. Together with data from human, mouse, zebrafish and yeast, our findings support a conserved role for ClC-3 in fluoride transport across eukaryotes. Furthermore, the various ClC-3 transcripts differ in fluoride-transport capacity. Human ClC-3 isoform b displays higher fluoride selectivity than isoforms c and X1. These three isoforms differ in their N- and C-terminal sequences, leading to distinct subcellular localizations. The extended N-terminus of isoform b appears to enhance fluoride transport, whereas the shorter N- and C-termini of isoform c promote its residence at the plasma membrane, thereby limiting harmful fluoride influx.

**Figure 9:**
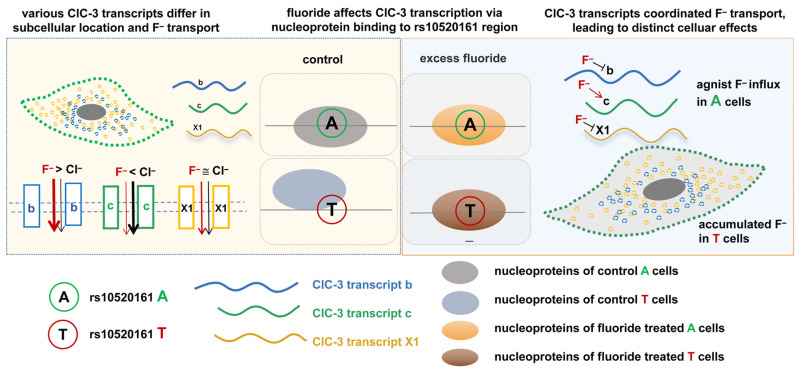
Schematic mechanisms by which rs10520161 is involved in dental fluorosis development. The rs10520161 A and T alleles exhibited different binding efficiencies with control and fluoride-treated nuclear extracts. This differential binding may modulate ClC-3 transcription, leading to altered mRNA levels of several isoforms involved in fluoride transport. In the left panel, red and black arrows indicate the directions and relative extents of fluoride and chloride transport, respectively. Colored wavy lines represent different ClC-3 transcripts (blue, transcript b; green, transcript c; yellow, transcript X1). In the right panel, red arrows indicate promotion, whereas black T-shaped lines indicate inhibition.

Next, fluoride might influence the binding of nuclear proteins to the rs10520161 region, indicating that the rs10520161 region directly modulates ClC-3 transcription, a role supported by the distinct fluoride-induced changes in the abundance of different ClC-3 transcripts. Cells may therefore coordinate ClC-3 transcript levels—for example, down-regulating fluoride-sensitive channels (b and X1) and up-regulating the fluoride-resistant channel (c)—to counteract the harmful effects of excess fluoride (Figure 9).

Previous studies have revealed the diverse biological and physiological roles of mammalian ClC-3, including the regulation of osteoclast, osteoblast and chondrocyte functions [30,31,32]. According to GeneHancer, rs10520161 is predicted to influence embryonic development in the craniofacial atlas. Consistently, in vivo knockdown of ClC-3 in zebrafish resulted in growth retardation and skeletal malformations. One new finding was that ClC-3 deficiency also impaired tooth formation and craniofacial patterning, effects similar to those observed in ClC-7 [16].

Excess fluoride disrupts tooth and bone development, and our mouse and zebrafish DF models further confirmed fluoride-associated pathologies such as cyst-like lesions and rough, unevenly calcified enamel or enameloid [15]. Although fluoride is used to treat osteoporosis [33,34] and partially rescued the craniofacial defects in *clcn3* morphants, the combination of *clcn3* deficiency and excess fluoride produced severe dental phenotypes, underscoring the protective role of ClC-3 against DF. Future work should clarify whether these divergent outcomes reflect the distinct roles of ClC-3 in tooth and bone tissues.

ClC-3 has been considered a CFTR-regulated ORCC molecule or its activator. ClC-3 interacts with CFTR, and both channel proteins associate with the Golgi PDZ proteins GOPC, EBP50 and PDZK1 [35,36]. Here, we show that both CFTR and ClC-3 mediate fluoride transport through distinct mechanisms. Together, these findings place the previously reported association between CFTR and ClC-3 in the context of DF development and raise the possibility that they act synergistically in this process. Further studies are warranted to test this hypothesis and elucidate the mechanistic basis of their potential interaction in DF.

Several limitations should be acknowledged. First, individual-level fluoride exposure indicators, including urinary fluoride concentration, dietary fluoride intake, and use of fluoride-containing toothpaste, were not collected and therefore could not be adjusted for in the analyses. Second, population stratification was not formally assessed using principal component analysis or ancestry-informative markers. Although all participants were ethnic Han Chinese, subtle genetic heterogeneity and potential population stratification cannot be completely excluded. Third, an independent replication cohort in additional high-fluoride populations is warranted, and the family-based analysis was limited by its relatively small sample size.

Despite these limitations, the consistency of the genetic association, pedigree, and functional data provides converging evidence supporting the involvement of the identified genes in dental fluorosis susceptibility. Future studies incorporating larger cohorts, more comprehensive fluoride exposure assessment, and additional mechanistic validation experiments will help further strengthen these findings.

## 5. Conclusions

Both *CFTR* and *CLCN3* are susceptibility genes for DF. Specific variants in these genes alter fluoride transport through distinct mechanisms. The coding variant M470V in *CFTR* directly impairs fluoride transport, whereas noncoding SNPs, such as rs10520161 in *CLCN3*, may influence alternative splicing isoforms of the *CLCN3* gene, which exhibit differential fluoride transport and may function collectively to mitigate the effects of excess fluoride exposure. Thus, DF arises from synergistic variants in *CFTR* and *CLCN3*. The interplay of these factors defines DF as a complex trait driven by ion channel dysregulation and highlights the multifactorial, gene-directed regulation of fluoride handling.

## Figures and Tables

**Table 1:** Variants distribution in *CFTR* and *CLCN3*.

Gene	SNP ID	Chr Position	Location	Ref/Alt	Case Genotype	Control Genotype	Alt Allele Frequency Case/Control	Allelic χ^2^	Allelic P	BH q	Allelic OR (95% CI)	Genotypic P
*CFTR*	rs213950	chr7:117559479	Exon	G/A	279 GG/407 GA/148 AA	585 GG/242 GA/62 AA	0.421/0.206	186.977	1.45 × 10^−42^	5.81 × 10^−42^	2.81 (2.42–3.27)	1.12 × 10^−40^
*CLCN3*	rs9996873	chr4:169635695	Intron	G/T	34 GG/246 GT/549 TT	22 GG/227 GT/633 TT	0.811/0.846	7.71	5.49 × 10^−3^	1.10 × 10^−2^	0.78 (0.65–0.93)	2.16 × 10^−2^
	rs17659581	chr4:169638289	Intron	T/C	169 TT/435 TC/229 CC	168 TT/415 TC/303 CC	0.536/0.576	5.615	1.78 × 10^−2^	1.78 × 10^−2^	0.85 (0.74–0.97)	1.04 × 10^−2^
	rs10520161	chr4:169662514	Intron	A/T	584 AA/247 AT/3 TT	673 AA/215 AT/1 TT	0.152/0.122	6.415	1.13 × 10^−2^	1.51 × 10^−2^	1.29 (1.06–1.56)	2.05 × 10^−2^

Note: Allelic χ^2^ and *p* values were calculated using the allelic association test (df = 1). ORs and 95% CIs were estimated for the alternative allele relative to the reference allele. Genotypic *p* values were calculated using the 3 × 2 genotype-based chi-square test (df = 2). BH q values were calculated using the Benjamini–Hochberg method for FDR correction. Base pair position is indicated according to the human reference sequence GRCh38.p14 (GCF_000001405.40): *CFTR*: NC_000007.14 (117480025..117668665) or *CLCN3*: NC_000004.12 (169620578..169723673).

## Data Availability

The original contributions presented in this study are included in the article/Supplementary Materials. Further inquiries can be directed to the corresponding author.

## Supplementary Materials

The following supporting information can be downloaded at: https://www.mdpi.com/article/10.3390/biom16070982/s1, Figure S1: GEF1 is necessary to against fluoride entrance in yeast; Table S1: Population information; Table S2: Information of Tag SNPs and related primers in microAssay; Table S3: Hardy–Weinberg equilibrium (HWE) analysis; Table S4: Multivariate Conditional Logistic Regression Analysis for Dental Fluorosis; Table S5: Relationship between DF Phenotype and rs213950 genotype; Table S6: Comparison of ClC-3 transcripts in human, mouse and zebrafish.
