# Supplementary material for: CFTR and ClC-3 Transport Fluoride Differently and Cause Dental Fluorosis in Different Ways
Source: Biomolecules. 2026 Jul 3;16(7):982. doi: 10.3390/biom16070982 (PMC13406507; doi:10.3390/biom16070982)
Supplement: Supplementary file 1 [file biomolecules-16-00982-s001.zip › biomolecules-4335798-supplementary.pdf]

Article

CFTR and ClC-3 Transport Fluoride Differently and Cause Dental Fluorosis in Different Ways

Yanli Zhang †, Songya Mao †, Xuan Wen, Zhenxia Liu, Ying Hao and Xiaohong Duan *

State Key Laboratory of Oral & Maxillofacial Reconstruction and Regeneration, National Clinical Research Center for Oral Disease, Shaanxi Key Laboratory of Stomatology, Department of Oral Biology, Clinic of Oral Rare Diseases and Genetic Diseases, School of Stomatology, The Fourth Military Medical University,
Xi'an 710032, China; yanlizhang@fmmu.edu.cn (Y.Z.); sy.mao@foxmail.com (S.M.); 15009261059@163.com (X.W.); liuzhx0716@126.com (Z.L.); haoying7ice@163.com (Y.H.)

* Correspondence: xhduan@fmmu.edu.cn; Tel.: +86-29-84776169

† These authors contributed equally to this work.

Supplementary Materials list

1. Table S1: Population information
2. Table S2: Information of Tag SNPs and related primers in microAssay
3. Table S3: Hardy–Weinberg equilibrium (HWE) analysis
4. Table S4: Multivariate Conditional Logistic Regression Analysis for Dental Fluorosis
5. Table S5: Relationship between DF Phenotype and rs213950 genotype
6. Table S6: Comparison of ClC-3 transcripts in human, mouse and zebrafish
    (1) Comparison of human ClC-3 transcripts
    (2) Comparison of mouse ClC-3 transcripts
    (3) Comparison of zebrafish ClC-3 transcripts
7. Figure S1 and Related Materials and Methods: GEF1 is necessary to against fluoride entrance in yeast.

1.Table S1

Table S1 Population information						
	**Control**	**DF**				**Total**
		total(n,%)	mild (n,%)	moderate(n,%)	severe(n,%)	
Male (n,%)	462(55.40)	447(50.28)	210(46.98)	142(31.77)	95(21.25)	909
Female(n,%)	372(44.60)	442(49.72)	212(47.96)	134(30.32)	96(21.72)	813
Total(n,%)	834(48.40)	889(51.60)	422(47.47)	276(31.05)	191(21.48)	1723

2. Table S2

Table S2 Information of Tag SNPs and related primers in microAssay				
**Gene**	**SNP_ID**	**2nd-PCRP**	**1st-PCRP**	**UEP_SEQ**
CFTR	rs213950	ACGTTGGATGCTCTGAAGGCTCCAGTTCTC	ACGTTGGATGGATGGGTTTATTTCCAGAC	CCAGTTCTCCCATAATCA
CFTR	rs214167	ACGTTGGATGGAGAGGGTATTCCAGGATGC	ACGTTGGATGTTTTGCCCCTCAGCTTTTTC	AGACAGGGAGATTATGGAT
CFTR	rs2237721	ACGTTGGATGTGGTCCTCAAACTCAAGAAC	ACGTTGGATGGGGAGCAGTATGTAGACAC	TGAAACTTAAATCTTGAGTCA
CFTR	rs121909001	ACGTTGGATGATGCTTTGATGACGCTTCTG	ACGTTGGATGTATGCCTGGCACCATTAAAG	CTATATTCATCATAGGAAACACC
CLCN1	rs1023252	ACGTTGGATGTGCTCTTCACTCCGTACCAC	ACGTTGGATGGAAATTCTCTCATCTAATGC	cctgCTCCACCTGCGTAGG
CLCN1	rs17164250	ACGTTGGATGTGTTCCGTTTTTGCCTTTCC	ACGTTGGATGTGGCAAAGAGGATGTACTTG	cccgTGCCTTTCCTTAACAATAAC
CLCN1	rs2367941	ACGTTGGATGGAAAAAGATTCTCTTCACAC	ACGTTGGATGAGTGCCCCATGGGATTAGAG	caacAAGATTCTCTTCACACAAAAAA
CLCN1	rs2103193	ACGTTGGATGCTACAGGACTTTTCAAAATC	ACGTTGGATGCCTCTAGACTGAGCTATACG	AAAATCTTGATATTTTAAAGGTACATA
CLCN3	rs10520161	ACGTTGGATGTTTTGTTCTTCCTCACAGGG	ACGTTGGATGTGGAATTGATGGAGACAGG	TCACAGGGAAGCTGAT

			C	
CLCN3	rs17659581	ACGTTGGATGATGCAGGGACTTCAGAATAC	ACGTTGGATGTTGACCTATTTCCTGGTGAG	CTTCAGAATACTTTACCTCTTT
CLCN3	rs9996873	ACGTTGGATGGAATGAAGAATTTCTCCAG	ACGTTGGATGACATACACAAGCAAAAATG	AATGAAGAATTTCTCCAGGTAGAT
CLCN4	rs10803414	ACGTTGGATGGACACCACCAGGGTCTTCC	ACGTTGGATGATTTGGAGGAGGGGACTCAT	TCCCTTCAGGCCTCC
CLCN4	rs17255432	ACGTTGGATGAGAGAACTCGTGATCTCAGC	ACGTTGGATGCTGAGCTGTCTCAGAGACTT	CAGCCGGATGGTTGG
CLCN4	rs2240018	ACGTTGGATGTCCTCTTCCAGAACCAAAGG	ACGTTGGATGGTTAAAACCCGATTCAGAAGG	CCAAAGGCTTTAGACATTTGTAG
CLCN4	rs2073938	ACGTTGGATGGGGAGGAGCAGATATATTGG	ACGTTGGATGCCACAATCCCTCCATATGAC	ggatGAGCAGATATATTGGCAGATC
CLCN5	rs6651707	ACGTTGGATGACATACTTCCCCATTCCACC	ACGTTGGATGAGTGCAGAGATAATAAGTG	CCCATTCCACCTGTAACCTC
CLCN5	rs34173954	ACGTTGGATGCCAGGGTCTTATGAGCAAAC	ACGTTGGATGATCTATGATGCCCACATCCG	gagaAAACTCTTCTTTGGCTTCAAGAAA
CLCN6	rs2050265	ACGTTGGATGGAATAGCTAATAAGCGGCCC	ACGTTGGATGGTGGTCACCAAGATTCTTTC	GCGGCCCTTCTAATAC
CLCN6	rs17350396	ACGTTGGATGAAGACCAGAGCCTCTGCTCA	ACGTTGGATGACACGGTATCCTCGCTAGTT	TCACACCCAGGAGCAGAGTG
CLCN7	rs2235579	ACGTTGGATGGAAACATGGTCAGCCAGTAG	ACGTTGGATGACTGGCCTTTCCCCTGTTC	GTTCAAGGCATTGAACAC
CLCN7	rs2745001	ACGTTGGATGAGGAATAGCCTGAGAAGCAC	ACGTTGGATGCCACCCTCTCCATCACTCAA	AGTGGCGTTAGAGAGAAGTGC
CLCN7	rs11645645	ACGTTGGATGTGTTGGCAAAAACCACAGGG	ACGTTGGATGGCAATAAATACTGGTGGGAG	GGATGGGATTTCCTGTAAGAGAT
CLCNKB	rs2275166	ACGTTGGATGGACTTCCTCCTCCCTTCCG	ACGTTGGATGAGGTTGTGACCTCCACAGAC	CCTGTGCTCTCCACCAGGGGATACT
CLCNKB	rs2015352	ACGTTGGATGCTCTCACCTCGGATGCCTC	ACGTTGGATGTCAGGGAACCCTGTGACTCT	CTCTCACCTCGGATGCCTCGGCGGATG

3. Table S3 Hardy–Weinberg equilibrium (HWE) analysis

Gene	SNP_ID	χ2	HWE(P)	Note
CFTR	rs213950	0.0008	0.98	
CFTR	rs214167	3.44	0.064	
CFTR	rs2237721	0.021	0.88	
CFTR	rs121909001	2.28	0.13	
CLCN1	rs1023252	1.28	0.26	
CLCN1	rs17164250	0.20	0.65	
CLCN1	rs2367941	0.45	0.50	
CLCN1	rs2103193	0.037	0.85	
CLCN3	rs10520161	18.85	1.4×10^{-5}	
CLCN3	rs17659581	2.07	0.15	
CLCN3	rs9996873	0.94	0.33	
CLCN4	rs10803414	N/A	N/A	***CLCN4*** and ***CLCN5*** are located on the X chromosome, conventional autosomal HWE assumptions are not applicable in the combined male and female population; therefore, these loci were excluded from HWE-based evaluation and subsequent analyses.
CLCN4	rs17255432			
CLCN4	rs2240018			
CLCN4	rs2073938			
CLCN5	rs6651707			
CLCN5	rs34173954			
CLCN6	rs2050265	0.30	0.58	
CLCN6	rs17350396	550	10^{-50}	
CLCN7	rs2235579	1.39	0.24	
CLCN7	rs2745001			
CLCN7	rs11645645	0.842	0.359	
CLCNKB	rs2275166	0.44	0.51	
CLCNKB	rs2015352	0.30	0.58	

4. Table S4

Multivariate Conditional Logistic Regression Analysis for Dental Fluorosis		
	**OR (95% CI)**	**P value**
**allele frequency**		
CFTR：rs213950（G）	1.000	-
CFTR：rs213950（A）	2.76（2.38-3.21）	3.12E-41
CLCN3：rs9996873（G）	1.000	-
CLCN3：rs9996873（T）	0.79（0.66-0.94）	6.21E-3
CLCN3：rs17659581（T）	1.000	-
CLCN3：rs17659581（C）	1.17（1.02-1.34）	2.01E-2
CLCN3：rs10520161（A）	1.000	-
CLCN3：rs10520161（T）	0.77（0.63-0.94）	1.32E-2
**gender**		
Female	1.000	-
Male	1.08（0.92-1.27）	3.56E-1
**residence**		
residence A	1.000	-
residence B	1.12（0.90-1.39）	3.15E-1
residence C	1.25（1.04-1.51）	2.24E-2

5. Table S5

Table S5 Relationship between DF Phenotype and rs213950 genotype					
Part 1 DF and rs213950					
Group	AA	GA	GG	χ^2 value	P value
control	148	407	279	183.975	P=1.12E-40
DF	62	242	585		
Part 2 DF degree and rs213950					
Genotype	control	DF degree		Chi-Square	P value
		mild	Medium	Severe		
AA	148	23	30	9	198.722	P= 7.97E-43
GA	407	99	88	55		
GG	279	300	158	127		

6. Table S6 : Comparison of ClC-3 transcripts in human, mouse and zebrafish

(1) Comparison of human ClC-3 transcripts								
**TanID**	**Protein**	**length**	**Isoform**	**Sequence**				
				**N-terminus**	**Middle 1**	**Middle 2**	**Middle 3**	**C-terminus**
NM_0012<br>43372.2	NP_0012<br>30301.1	791	a	**MESEQLFHRGYYR<br>NSYNSITSASSDEEL<br>LDGAGVIMDFQTS<br>EDDNLLDGDTAV**	GTHYTMTNGGSINSS<br>THLLDLLDEPIPGVGT<br>YDDFHTIDWVREKCK<br>DRERHRRINSKKKES<br>AWEMTKSLYDAWSG<br>WLVVTLTGLASGALA<br>GLIDIAADWMTDLKE<br>GICLSALWYNHEQCC<br>WGSNETTFEERDKCP<br>QWKTWAELIIGQAEG<br>PGSYIMNYIMYIFWAL<br>SFAFLAVSLVKVFAPY<br>ACGSGIPEIKTILSGFII<br>RGYLGKWTLMIKTITL<br>VLAVASGLSLGKEGP<br>LVHVACCCGNIFSYL<br>FPKYSTNEAKKREV	—	SYYFPLKTLWRSFFAA<br>LVAAFVLRSINPFGNS<br>RLVLFYVEYHTPWYL<br>FELFPFILLGVFGGLW<br>GAFFIRANIAWCRRR<br>KSTKFGKYPVLEVIIV<br>AAITAVIAFPNPYTRL<br>NTSELIKELFTDCGPL<br>ESSSLCDYRNDMNAS<br>KIVDDIPDRPAGIGVY<br>SAIWQLCLALIFKIIM<br>TVFTFGIKVPSGLFIPS<br>MAIGAIAGRIVGIAVE<br>QLAYYHHDWFIFKE<br>WCEVGADCITPGLYA<br>MVGAAACLGGVTR<br>MTVSLVVIVFELTGGL<br>EYIVPLMAAVMTSK<br>WVGDAFGREGIYEA<br>HIRLNGYPFLDAKEE<br>FTHTTLAADVMRPRR<br>NDPPLAVLTQDNMT<br>VDDIENMINETSYNG<br>FPVIMSKESQRLVGFA<br>LRRDLTIAIESARKKQ	**DILRHMAQTANQD<br>PASIMFN**

							EGIVGSSRVCFAQHT<br>PSLPAESPRPLKLRSIL<br>DMSPFTVTDHTPMEI<br>VVDIFRKLGLRQCLV<br>THNGRLLGIITKK	
NM_0018<br>29.4	NP_0018<br>20.2	818	b	**MESEQLFHRGYYR**<br>**NSYNSITSASSDEEL**<br>**LDGAGVIMDFQTS**<br>**EDDNLLDGDTAV**	As above	LSAASAAGVSVAF<br>GAPIGGVLFSLEEV	As above	**DILRHMAQTANQD**<br>**PASIMFN**
NM_0012<br>43374.2	NP_0012<br>30303.2	791	c	MDASSDPYLPYDGG<br>GDSIPLRELHKR	As above	As above	As above	**DILRHMAQTANQD**<br>**PASIMFN**
NM_1738<br>72.4	NP_7762<br>97.2	866	e	**MESEQLFHRGYYR**<br>**NSYNSITSASSDEEL**<br>**LDGAGVIMDFQTS**<br>**EDDNLLDGDTAV**	As above	As above	As above	NILEHLEQLKQHVE<br>PLAPPWHYNKKRYP<br>PAYGPDGKPRPRFN<br>NVQLNLTDEEREET<br>EEEVYLLNSTTL
XM_00526<br>2726.3	XP_0052<br>62783.1	839	X1	MDASSDPYLPYDGG<br>GDSIPLRELHKR	As above	As above	As above	NILEHLEQLKQHVE<br>PLAPPWHYNKKRYP<br>PAYGPDGKPRPRFN<br>NVQLNLTDEEREET<br>EEEVYLLNSTTL
XM_01153<br>1586.2	XP_0115<br>29888.1	831	X2	**MNLNDEDHHFTSL**<br>**EIDHR**	As above	As above	As above	NILEHLEQLKQHVE<br>PLAPPWHYNKKRYP<br>PAYGPDGKPRPRFN<br>NVQLNLTDEEREET<br>EEEVYLLNSTTL
NM_0018<br>29.4 mut*		818	CLCN3b<br>MUT	**MESEQLFHRGYYR**<br>**NSYNSITSASSDEEL**<br>**LDGAGVIMDFQTS**<br>**EDDNLLDGDTAV**	GTHYTMTNGGSINSS<br>TH**AADAA**DEPIPGVG<br>TYDDFHTIDWVREKC<br>KDRERHRRINSKKKES	As above	As above	**DILRHMAQTANQD**<br>**PASIMFN**

AWEMTKSLYDAWSG<br>WLVVTLTGLASGALA<br>GLIDIAADWMTDLKE<br>GICLSALWYNHEQCC<br>WGSNETTFEERDKCP<br>QWKTWAELIIGQAEG<br>PGSYIMNYIMYIFWAL<br>SFAFLAVSLVKVFAPY<br>ACGSGIPEIKTILSGFII<br>RGYLGKWTLMIKTITL<br>VLAVASGLSLGKEGPL<br>VHVACCCGNIFSYLFP<br>KYSTNEAKKREV

(2) Comparison of mouse ClC-3 transcripts

TanID	Protein	length	Isoform	Sequence		
				**N-terminus**	**Middle**	**C-terminus**
NM_007711.4	NP_031737.1	760	a	–	MTNGGSINSSTHLLDLLDEPIPGVGTYDDFHTIDWVREKCKDRER<br>HRRINSKKKESAWEMTKSLYDAWSGWLVVTLTGLASGALAGLI<br>DIAADWMTDLKEGICLSALWYNHEQCCWGSNETTFEERDKCPQ<br>WKTWAELIIGQAEGPGSYIMNYIMYIFWALSFAFLAVSLVKVFAP<br>YACGSGIPEIKTILSGFIIRGYLGKWTLMIKTITLVLAVASGLSLGKE<br>GPLVHVACCCGNIFSYLFPKYSTNEAKKREVLSAASAAGVSVAF<br>GAPIGGVLFSLEEVSYYFPLKTLWRSFFAALVAAFVLRSINPFGNS<br>RLVLFYVEYHTPWYLFELFPFILLGVFGGLWGAFFIRANIAWCRR<br>RKSTKFGKYPVLEVIIVAAITAVIAFPNPYTRLNTSELIKELFTDCG<br>PLESSSLCDYRNDMNASKIVDDIPDRPAGVGVYSAIWQLCLALIF<br>KIIMTVFTFGIKVPSGLFIPSMAIGAIAGRIVGIAVEQLAYYHHDW<br>FIFKEWCEVGADCITPGLYAMVGAAACLGGVTRMTVSLVVIVFE<br>LTGGLEYIVPLMAAVMTSKWVGDAFGREGIYEAHIRLNGYPFLD<br>AKEEFTHTTLAADVMRPRRSDPPLAVLTQDNMTVDDIENMINE	**RLLGIITKKDILRH<br>MAQTANQDPASIM<br>FN**

					TSYNGFPVIMSKESQRLVGFALRRDLTIAIESARKKQEGIVGSSRV<br>CFAQHTPSLPAESPRPLKLRSILDMSPFTVTDHTPMEIVVDIFRKL<br>GLRQCLVTHNG	
NM_1738<br>73.2	NP_7762<br>98.1	818	b	**MESEQLFHRGYYR<br>NSYNSITSASSDEEL<br>LDGAGAIMDFQTS<br>EDDNLLDGDTAAG<br>THYT**	As above	**RLLGIITKKDILRH<br>MAQTANQDPASIM<br>FN**
NM_1738<br>76.4	NP_7763<br>01.1	791	c	MDASSDPYLPYDGG<br>GDSIPLRELHKRGT<br>HYT	As above	**RLLGIITKKDILRH<br>MAQTANQDPASIM<br>FN**
NM_1738<br>74.2	NP_7762<br>99.1	866	e	**MESEQLFHRGYYR<br>NSYNSITSASSDEEL<br>LDGAGAIMDFQTS<br>EDDNLLDGDTAAG<br>THYT**	As above	IVLGIITKKNILEHLE<br>QLKQHVEPLTPPWH<br>YNKKRYPPSYGPDG<br>KPRPRFNNVQLSPV<br>DEDREETEEEVRLLN<br>STIL
NM_0014<br>16732.1	NP_0014<br>03661.1	839	g	MDASSDPYLPYDGG<br>GDSIPLRELHKRGT<br>HYT	As above	IVLGIITKKNILEHLE<br>QLKQHVEPLTPPWH<br>YNKKRYPPSYGPDG<br>KPRPRFNNVQLSPV<br>DEDREETEEEVRLLN<br>STIL
NM_0014<br>16733.1	NP_0014<br>03662.	808	h	–	As above	IVLGIITKKNILEHLE<br>QLKQHVEPLTPPWH<br>YNKKRYPPSYGPDG<br>KPRPRFNNVQLSPV<br>DEDREETEEEVRLLN<br>STIL
NM_0014	NP_0014	760	i	–	As above	RLLGIITKKDILRHM

TanID	Protein	length	Isoform	N-terminus	Middle	C-terminus
16734.1	03663.1					AQTANQDPASIMFN
(3) Comparison of zebrafish ClC-3 transcripts						
**NC_007118.7 Reference GRCz11 Primary Assembly Range 7511869..7642050**						
**TanID**	**Protein**	**length**	**Isoform**	**Sequence**		
				**N-terminus**	**Middle**	**C-terminus**
**XM_0214 77689.1**	**XP_0213 33364.1**	874	X1	meseqlfnrgygrnsynsit sassdeelldgagvimdfht teddnlldgdasp	gsnyamsngggggggasssthlldlleepipgvgtyddfhtidwvrekckdrerhrkinsk kkesaweftkslydawsgwlvvtltglasgalaggidiaadwmndlkegvclsamwfnh eqccwgsnkttfaerdkcpqwktwaelilgqeqgpgsyimnyfmftfwalsfaflavslvk vfapyacgsgipeiktilsgfiirgylgkwtlmiktitlvlavasglslgkegplvhvacccgnif sylfpkyskneakkrevlsaasaagvsvafgapiggvlfsleevsyyfplktlwrsffaalvaa fvlrsinpfgnsrlvlfyveyhtpwylfelfpfillgvfgglwgaffiraniawcrrrkstrfgky pvlevitvaaitaivafpnpytrqntselikelftdcgplessqlcqyrslmngsqadptgpdt asaatpgvysamwqlslalvfkiimtiftfglkvpsglfipsmaigaiagrivgiaveqlayyh hdwfvfrewcevgadcitpglyamvgaaaclggvtrmtvslvvivfeltggleyivplmaa vmtskwvgdafgregiyeahirlngypfldakeefthttlarevmrprrsdpplavltqdd mtlaelqgiisetsyngfpvivskesqrlvgfalrrditiaienarrkqegivlnsrvyftqhaptl padsprplklrsildmspftvtdhtpmeivvdifrklglrqclvthng	ivlgiitkknilehleeikqhv epltppwyyykkryppsy gpdgkprprvhnvqlapss shyqedeieeevrlldnssl
**XM_0214 77690.1**	XP_0213 33365.1	849	X2	meeesadpylpydggggd tiplqelsgr	As above	ivlgiitkknilehleeikqhv epltppwyyykkryppsy gpdgkprprvhnvqlapss shyqedeieeevrlldnssl
**XM_0214 77691.1**	XP_0213 33366.1	824	X3	meseqlfnrgygrnsynsit sassdeelldgagvimdfht teddnlldgdasp	As above	ivlgiitkkknilehleeikqh vepliddi
**XM_0214 77692.1**	**XP_0213 33367.1**	824	X3?	meseqlfnrgygrnsynsit sassdeelldgagvimdfht teddnlldgdasp	As above	ivlgiitkknilehleeikqhv epliddi
**XM_0214 77693.1**	XP_0213 33368.1	824	X4	meseqlfnrgygrnsynsit sassdeelldgagvimdfht teddnlldgdasp	As above	rllgiitkkdilrhmaqman qdpesimfn

**XM_021477694.1**	XP_021333369.1	799	X5	meeesadpylpydggggd tiplqelsgr	As above	rllgiitkkdilrhmaqman qdpesimfn
**Reference GRCz11 ALT_DRER_TU_1 NW_018394672.1 Reference GRCz11 ALT_DRER_TU_1 Range 62642..173786**						
**XM_005166345.4**	XP_005166402.1	874	X1	meseqlfnrgygrnsynsit sassdeelldgagvimdfht teddnlldgdasp	gsnyamsngggggggasssthlldlleepipgvgtyddfhtidwvrekckdrerhrkinsk<br>kkesaweftkslydawsgwlvvtltglasgalaggidiaadwmndlkegvclsamwfnh<br>eqccwgsnkttfaerdkcpqwktwaelilgqeqgpgsyimnyfmftfwalsfaflavslvk<br>vfapyacgsgipeiktilsgfiirgylgkwtlmiktitlvlavasglslgkegplvhvacccgnif<br>sylfpkyskneakkrevlsaasaagvsvafgapiggvlfsleevsyyfplktlwrsffaalvaa<br>fvlrsinpfgnsrlvlfyveyhtpwylfelfpfillgvfgglwgaffiraniawcrrrkstrfgky<br>pvlevitvaaitaivafpnpytrqntselikelftdcgplessqlcqyrslmngsqadptgpdt<br>asaatpgvysamwqlslalvfkiimtiftfglkvpsglfipsmaigaiagrivgiaveqlayyh<br>hdwfvfrewcevgadcitpglyamvgaaaclggvtrmtvslvvivfeltggleyivplmaa<br>vmtskwvgdafgregiyeahirlngypfldakeefthttlarevmrprrsdpplavltqdd<br>mtlaelqgiisetsyngfpvivskesqrlvgfalrrditiaienarrkqegivlnsrvyftqhaptl<br>padsprplklrsildmspftvtdhtpmeivvdifrklglrqclvthng	ivlgiitkknilehleeikqhv epltppwyyykkryppsy gpdgkprprvhnvqlapss shyqedeieeevrlldnssl
**XM_001923468.7**	XP_001923503.1	874	X1	meseqlfnrgygrnsynsit sassdeelldgagvimdfht teddnlldgdasp	As above	ivlgiitkknilehleeikqhv epltppwyyykkryppsy gpdgkprprvhnvqlapss shyqedeieeevrlldnssl
**XM_003198891.5**	XP_003198939.1	849	X2	meeesadpylpydggggd tiplqelsgr	As above	ivlgiitkknilehleeikqhv epltppwyyykkryppsy gpdgkprprvhnvqlapss shyqedeieeevrlldnssl
**XM_005166349.4**	XP_005166406.1	824	X3	meseqlfnrgygrnsynsit sassdeelldgagvimdfht teddnlldgdasp	As above	ivlgiitkknilehleeikqhv epliddi
**XM_009302926.3**	XP_009301201.1	824	X3	meseqlfnrgygrnsynsit sassdeelldgagvimdfht teddnlldgdasp	As above	ivlgiitkknilehleeikqhv epliddi
**XM_0051**	XP_0051	824	X4	meseqlfnrgygrnsynsit	As above	rllgiitkkdilrhmaqman

**66348.4**	66405.1			sassdeelldgagvimdfht teddnlldgdasp		qdpesimfn
**XM_0051 66350.4**	XP_0051 66407.1	799	X5	meeesadpylpydggggd tiplqelsgr	As above	rllgiitkkdilrhmaqman qdpesimfn

* This study. The mutant amino acid was highlighted in red.

7. Figure S1 and Related Materials and Methods

7.1. Results

The yeast only have one voltage gated chloride channel, GEF1, similar to human *CLCN3* gene[3]; thus, we used yeast model to observe the effect of voltage gated chloride channel against overdose fluoride. Based on S. cerevisiae ΔGEF1 with the deletion of GEF1, we set up two rescued lines with overexpressing human *CLCN3* transcript b (NP_001820.2) (ΔGEF1/CLCN3 WT) and *CLCN3* b mutant (NP_001820.2 p.LLDLL 71~75 AADAA) (ΔGEF1/CLCN3 MUT), respectively. When the four strains were cultured on fluoride-free solid agar, they grew at comparable rates. In contrast, on medium supplemented with 50 mM NaF, their growth rates diverged markedly.

When the four strains were cultured on fluoride-free solid agar, they grew at comparable rates. In contrast, on medium supplemented with 50 mM NaF, their growth rates diverged markedly: growth of ΔGEF1 strain was severely inhibited, rescued strain ΔGEF1/CLCN3 WT regained fluoride tolerance, and the mutant strain (ΔGEF1/CLCN3 MUT) showed partial rescue (**Figure S1**).

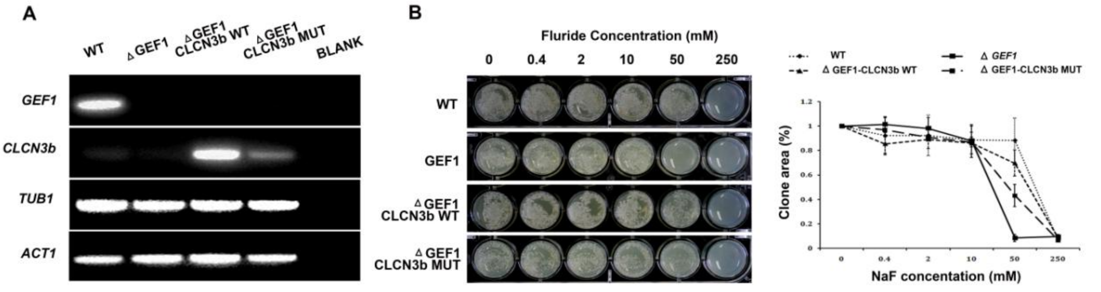

Figure S1. GEF1 is necessary to against fluoride entrance in yeast. (**A**) RT-PCR electropherogram. Wildtype S. cerevisiae expressed *GEF1* while the ΔGEF1 strain did not. *ΔGEF1/CLCN3b* WT had a higher amount of *CLCN3b* than the ΔGEF1/*CLCN3b* MUT. (**B**) Effect of fluoride on the growth characteristics of four strains. On solid media containing 50mM fluoride, the growth of ΔGEF1 was significantly inhibited. The wild type *CLCN3* strain tolerated the high fluoride concentrations, while the mutant one (*ΔGEF1*/*CLCN3* MUT) only rescued part of activity.

7.2. Material and Methods

7.2.1. Stains

The yeast genome encodes GEF1 that has amino acid homology to the CLC voltage-gated chloride channel superfamily. The yeast (*Saccharomyces cerevisiae*) strain RGY30 (diploid of wild type (2a)) and RGY147 (diploid with gef1:leu2 and gef1:His3 (2a)) were kindly provided by Dr. Roberto Gaxiola in Arizona State University. The wild type and mutant full length cDNA of human *CLCN3b* was synthesized and subcloned into pADH1 vector. Then the vectors were transformed into RGY147 and the new yeast strains containing wild type and mutant *CLCN3* were generated. The mutant sites locate in c.770~784 of NM_001829 c.DNA (tta ctg gat ctt ttg→ gca gcg gat gct gcg). The stains were cultured in the selective YPD medium as required.

7.2.2. Rescue and detection of over expression

The mRNA of yeast strains were extracted and cDNA was synthesized as the regular methods. The expression of *CLCN3* and *GEF1* were detected by regular RT-PCR, and *TUB1* and *ACT1* were used as the internal control.

7.2.3. Effect of fluoride on the growth rate

The yeast were amplified in selective YPD medium to OD600 value around 0.4. 50 μL yeast suspension (OD600=0.4) were seeded in 24 well plate containing YPD agar medium with different fluoride (0.4, 2, 10, 50, 250 mM NaF) and further cultured in 30ºC for 3 days. The plate was photographed and the clone area were calculated with NIH ImageJ. The experiment was repeated at least three times.
